# Dataset on ground radiometric survey in part of the Eastern Dahomey Basin, SW Nigeria

**DOI:** 10.1016/j.dib.2017.09.021

**Published:** 2017-09-18

**Authors:** Kehinde D. Oyeyemi, Ahzegbobor P. Aizebeokhai, Oluwarotimi M. Olofinnade

**Affiliations:** a Department of Physics, Covenant University, Ota, Nigeria; b Department of Civil Engineering, Covenant University, Ota, Nigeria

**Keywords:** SPEC RS-125, Radiometric survey, Natural radionuclides, Gamma-radiation dose rate, Southwestern Nigeria

## Abstract

The dataset for this article contains the measured activity concentration of radionuclides and gamma-radiation dose rate obtained from the radiometric survey in Ota, Ado-Odo Local Government Area, Ogun State, Nigeria. The data were manually collected in fifty (50) locations using the Super SPEC RS-125 spectrometer for about three (3) weeks in January, 2017. The descriptive statistical analysis of the data were equally explored for possible statistical relationships. The field dataset is made available publicly for further extended analyses that can provide insights into the safety status of the study area from radiological health concerns. The dataset could also serve as a significant baseline radiometric data for future epidemiology researches and monitoring initiative in Ota and its environs.

**Specifications Table**

**Table:** 

Subject area	Earth, Environment and Planetary Science
More specific subject area	Environmental Geophysics
Type of data	Tables and Figures
How data was acquired	Super SPEC RS-125 Spectrometer with large 2.0×2.0 Sodium-Iodide (NAI) Crystal
Data format	Raw, Analyzed
Experimental factors	The radiometric surveys were conducted with RS-125 spectrometer placed 1.0 metre above the soil covered.
Experimental features	Determination of the activity concentrations of the natural radionuclides such as Uranium-238 (^238^U), Thorium-232 (^232^Th) and Potassium-40 (^40^K). Gamma-radiation dose rate were equally determined.
Data source location	Ota, Ado-Odo Local Government Area (L.G.A.), Ogun State, Nigeria, The study area for the data acquisition is bounded by latitude 6°39'–6°41'N and longitude 3°11'–3°13'
Data accessibility	All the data are in this data article

**Value of the data**

• The dataset could be used to determine the concentration levels of the primordial naturally occurring radionuclides and evaluate the extent of radiation dose exposure within Ota and its environs.
• The dataset could be used to compute several health radiological hazards parameters such as the annual effective dose, radiation indices, excess lifetime cancer risks and annual gonadal dose equivalent. The health implications of these radiation hazards could also be evaluated by comparing them with the worldwide standard threshold limits.
• The ground radiometric survey can be replicated in other neighboring communities such as Sango, Atan, Owode, Idanyi and Igbesa. Also, the survey can be extended to cover the entire Ado-Odo L.G.A. or the entire Ogun state on a regional scale.
• The dataset could be used for educational purposes in applications of radiometric method, environmental radioactivity and pollution studies, and radiation physics. Similar data articles can be found in [1], [2], [3], [4], [5], [6], [7].
• Findings can be extended to other radionuclides not considered in this article

## 1 Data

The dataset contains the measured activity concentrations of naturally occurring radionuclides and gamma-ray dose rate for fifty (50) locations within Ota community, southwestern Nigeria. The radionuclides considered are Uranium-238 (^238^U), Thorium-232 (^232^Th) and Potassium-40 (^40^K ) as shown in Table 1 alongside the geographical coordinates of each measurement station point. When the activity concentration levels of these radionuclides and the radiation dose rate are higher than the minimum permissible limits, prolong exposure of the residents can cause severe health crisis such as sterility, atrophy, lung cancer, anaemia, leucopoenia and death. Furthermore, the descriptive analyses as presented in Table 2 were carried out to better understand the statistical distribution of the measured data. The correlation analysis was adopted using three different techniques as shown in Table 3 to reveal the presence of any causative relationships among the measured radiological parameters.

**Table 1:** The measured activity concentrations of radionuclides (Bq/kg) and gamma-ray dose rate (nGr/h).

Stations	Latitude	Longitude	^238^U (Bq/kg)	^232^Th (Bq/kg)	^40^K (Bq/kg)	Dose rate (nGr/h)
L1.	6°40'51.09"N	3°11'26.98"E	24.0825 ± 0.02	47.908 ± 0.24	391.25± 2.5	130.35 ± 2.05
L2.	6°40'46.75"N	3°11'30.12"E	31.4925 ± 0.03	47.908 ± 0.24	438.20 ± 2.8	141.9 ± 7.07
L3.	6°40'37.59"N	3°11'31.22"E	46.93 ± 0.04	51.562 ± 0.25	438.20 ± 2.8	166.1 ± 0.71
L4.	6°40'21.19"N	3°11'34.07"E	29.0225 ± 0.02	72.877 ± 0.36	485.15 ± 3.1	181.72 ±± 5.23
L5.	6°40'6.96"N	3°11'31.45"E	33.9625 ± 0.03	63.945 ± 0.32	516.45 ± 3.3	163.79 ± 7.57
L6.	6°39'54.72"N	3°11'32.43"E	38.9025 ± 0.03	53.592 ± 0.26	406.90 ± 2.6	151.91 ± 0.78
L7.	6°39'46.70"N	3°11'33.19"E	20.995 ± 0.02	58.261 ± 0.29	391.25 ± 2.5	144.43 ± 1.48
L8.	6°39'34.66"N	3°11'35.20"E	12.9675 ± 0.01	59.479 ± 0.29	391.25 ± 2.5	135.85 ± 5.87
L9.	6°39'35.97"N	3°11'54.55"E	11.7325 ± 0.01	59.682 ± 0.29	422.55 ± 2.7	136.51 ± 6.58
L10.	6°39'48.59"N	3°11'57.14"E	17.29 ± 0.014	56.231 ± 0.28	516.45 ± 3.3	146.96 ± 18.67
L11.	6°40'6.30"N	3°11'51.71"E	43.8425 ± 0.036	43.442 ± 0.21	328.65 ± 2.1	136.18 ± 3.25
L12.	6°40'20.46"N	3°11'51.15"E	30.875 ± 0.025	59.682 ± 0.29	359.95 ± 2.9	149.6 ± 6.08
L13.	6°40'27.81"N	3°11'44.76"E	24.0825 ± 0.02	73.08 ± 0.36	453.85 ± 2.9	171.93 ± 3.46
L14.	6°40'38.34"N	3°11'39.64"E	36.4325 ± 0.03	64.757 ± 0.32	469.50 ± 3.0	173.47 ± 5.02
L15.	6°40'46.34"N	3°11'38.36"E	22.8475 ± 0.02	69.02 ± 0.34	438.20 ± 2.8	163.02 ± 4.53
L16.	6°40'53.00"N	3°11'45.05"E	32.7275 ± 0.03	53.795 ± 0.27	547.75 ± 3.4	160.27 ± 10.39
L17.	6°40'52.88"N	3°12'8.17"E	22.23 ± 0.02	58.464 ± 0.29	532.10 ± 3.4	155.87 ± 4.88
L18.	6°40'42.82"N	3°12'7.77"E	31.4925 ± 0.03	39.991 ± 0.20	422.55 ± 2.7	128.15 ± 10.82
L19.	6°40'43.24"N	3°11'52.81"E	30.2575 ± 0.025	69.832 ± 0.34	438.20 ± 2.8	171.93± 4.74
L20.	6°40'32.32"N	3°12'5.80"E	17.29 ± 0.014	74.298 ± 0.37	532.10 ± 3.4	173.25 ± 9.69
L21.	6°40'27.97"N	3°11'58.14"E	27.17 ± 0.022	59.479 ± 0.29	453.85 ± 2.9	155.32 ± 6.22
L22.	6°40'23.43"N	3°12'3.53"E	27.7875 ± 0.023	55.013 ± 0.27	438.20 ± 2.8	148.28 ± 4.95
L23.	6°40'12.56"N	3°12'3.03"E	25.3175 ± 0.021	63.945 ± 0.32	594.70 ± 3.8	171.6 ± 0.14
L24.	6°40'11.63"N	3°12'13.40"E	31.4925 ± 0.026	42.63 ± 0.21	359.95 ± 2.3	125.95 ± 5.73
L25.	6°40'3.47"N	3°11'59.63"E	54.9575 ± 0.045	48.314 ± 0.24	344.30 ± 2.2	156.31 ± 1.48
L26.	6°39'55.38"N	3°12'2.92"E	28.405 ± 0.023	48.923 ± 0.24	516.45 ± 3.3	146.85 ± 3.89
L27.	6°39'42.19"N	3°12'5.36"E	48.165 ± 0.04	67.193 ± 0.33	406.90 ± 2.6	177.54 ± 17.82
L28.	6°39'36.66"N	3°12'8.02"E	25.231 ± 0.13	82.621 ± 0.41	453.85 ± 2.9	161.92 ± 10.04
L29.	6°39'37.76"N	3°12'22.62"E	51.87 ± 0.04	54.201 ± 0.27	359.95 ± 2.3	162.36 ± 5.52
L30.	6°39'37.86"N	3°12'28.59"E	19.1425 ± 0.02	60.088 ± 0.30	485.15 ± 3.1	151.03 ± 7.42
L31.	6°39'48.04"N	3°12'30.40"E	17.9075 ± 0.02	61.915 ± 0.31	359.95 ± 2.3	141.13 ± 11.38
L32.	6°40'7.79"N	3°12'29.80"E	9.2625 ± 0.01	61.712 ± 0.30	375.60 ± 2.4	134.64 ± 3.11
L33.	6°40'21.14"N	3°12'30.05"E	22.8475 ± 0.02	56.028 ± 0.28	469.50 ± 3.0	146.63 ± 4.17
L34.	6°40'35.79"N	3°12'30.71"E	65.455 ± 0,05	49.938 ± 0.25	547.75 ± 3.5	147.4 ± 4.24
L35.	6°40'45.17"N	3°12'30.89"E	12.35 ± 0.01	50.953 ± 0.25	422.55 ± 2.7	125.07 ± 0.21
L36.	6°40'57.43"N	3°12'29.75"E	41.3725 ± 0.03	34.104 ± 0.17	234.75 ± 1.5	111.32 ± 2.40
L37.	6°40'59.39"N	3°12'40.66"E	30.2575 ± 0.03	62.727 ± 0.31	422.55 ± 2.7	160.27 ± 5.59
L38.	6°40'52.27"N	3°12'37.74"E	48.7825 ± 0.04	38.976 ± 0.19	313.00 ± 2.0	134.97 ± 4.88
L39.	6°40'55.56"N	3°12'45.61"E	6.7925 ± 0.01	63.133 ± 0.31	469.50 ± 3.0	142.23 ± 7.00
L40.	6°41'2.44"N	3°12'55.26"E	20.3775 ± 0.02	41.006 ± 0.2	485.15 ± 3.1	123.86 ± 2.12
L41.	6°40'55.86"N	3°12'58.65"E	14.82 ± 0.01	53.592 ± 0.26	328.65 ± 2.1	122.87 ± 2.19
L42.	6°40'51.38"N	3°12'59.57"E	14.82 ± 0.01	51.765 ± 0.26	751.20 ± 4.8	138.71 ± 4.60
L43.	6°40'45.93"N	3°12'59.07"E	17.29 ± 0.01	51.562 ± 0.25	1032.90 ± 6.6	154.33 ± 3.89
L44.	6°40'44.09"N	3°12'40.31"E	13.585 ± 0.01	37.352 ± 0.18	1220.70 ± 7.8	132.33 ± 10.96
L45.	6°40'45.18"N	3°12'49.53"E	13.585 ± 0.01	67.396 ± 0.33	641.65 ± 4.1	154.88 ± 7.35
L46.	6°40'39.02"N	3°13'0.79"E	16.6725 ± 0.01	51.562 ± 0.25	892.05 ± 5.7	148.39 ± 5.59
L47.	6°40'35.16"N	3°13'6.92"E	16.6725 ± 0.01	73.689 ± 0.36	860.75 ± 5.5	179.41 ± 2.90
L48.	6°40'39.43"N	3°13'14.13"E	17.9075 ± 0.02	69.02 ± 0.34	657.30 ± 4.2	168.41 ± 4.03
L49.	6°40'46.49"N	3°13'4.77"E	14.82 ± 0.01	52.78 ± 0.26	688.60 ± 4.4	137.83 ± 7.14
L50.	6°40'44.85"N	3°13'13.41"E	15.4375 ± 0.01	49.735 ± 0.25	626.00 ± 4.0	133.21± 8.84

**Table 2:** Summary of the statistical analysis of the data.

Statistic	^238^U	^232^Th	^40^K	Dose-Rate
Mean	26.6002	56.7832	502.6780	149.5648
Standard error	1.83902	1.50074	25.9560	2.3833
Median	24.083	56.1295	453.85	148.3350
Standard deviation	13.0038	10.6119	183.5368	16.8521
Variance	169.100	112.612	33,685.741	283.994
Kurtosis	0.597	−0.241	4.985	−0.702
Skewness	0.968	0.065	2.033	0.006
Range	58.66	48.52	985.95	70.40
Minimum	6.79	34.10	234.75	111.32
Maximum	66.46	82.62	1220.70	181.72
Sum	1330.01	2839.16	25,133.90	7478.24

**Table 3:** A correlation matrix of the concentration of the radionuclides elements and gamma-ray dose for Pearson, Kendall tau and spearman correlation techniques.

Correlation Coefficient	Pearson				Kendall tau				Spearman rho			
Variables	^238^U	^232^Th	^40^K	Dose-Rate	^238^U	^232^Th	^40^K	Dose-Rate	^238^U	^232^Th	^40^K	Dose-Rate
^238^U	1				1				1			
^232^Th	−0.255	1			−0.141	1			−0.206	1		
^40^K	−0.385	0.005	1		−0.308	0.153	1		−0.385	0.204	1	
Dose-Rate	0.197	0.763	0.158	1	0.204	0.584	0.226	1	0.296	0.742	0.318	1

## 2 Experimental design, materials and methods

Radiation is an inevitable component of any natural environment. The agents producing radiation can be found in air, water, soil, sediments, food and in several other materials in the environment. Exposure of human to radiation varies from one geographical location to another due to the complexity of the earth formations and geology. Several radiogenic components analyses in air, water, soil, sediments, food and other materials have been carried out in Nigeria [8], [9], [10], [11], [12], [13], [14], [15], [16], [17], [18]. The variation in different geographical locations and the corresponding level of human exposure to radiation have also been reported. Geogenic sources of radiation involving different geological formations on the earth and anthropogenic sources of radiation due to the use of several man-made nuclear devices have been stated to be the responsible for natural environmental radioactivity.

### 2.1 Field survey

**Fig. 1:**
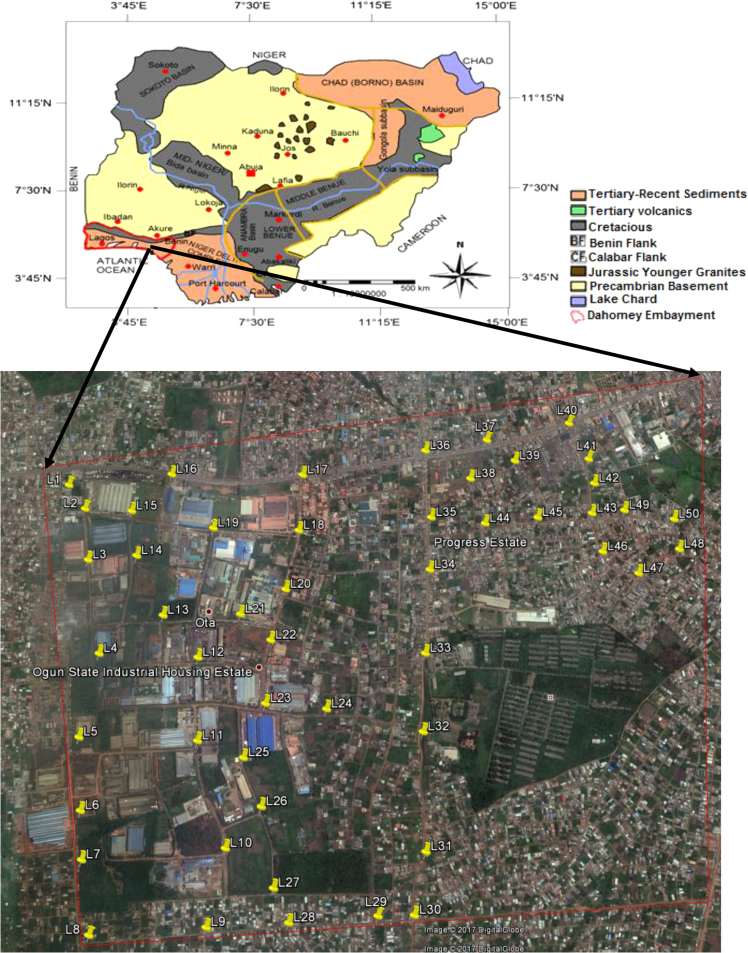
Geological map of Nigeria showing Dahomey Embayment (After [19]) and field basemap for the ground radiometric surveys with yellow marker showing each station point (Goggle Earth).

The study area is within the eastern portion of the Dahomey (Benin) Basin, southwestern Nigeria and the basemap showing all the station points for the radiometric survey is presented in Fig. 1. The data were manually collected during the dry season (January, 2017) for fifty station points using Super RS-125 spectrometer with 2.0×2.0 NAI crystal, which is a hand-held radiometric survey equipment for geophysical investigations. The equipment has good integrated design and large detector, large storage device and high sensitivity. The RS-125 spectrometer was calibrated by using a 5 min accumulation on Uranium, Thorium and Potassium pads with background pads having 10 min accumulation in accordance with the Canadian Geophysical Institute regulation. The preference for this equipment in this research is not unconnected to its high accuracy with probable error of five percent. During the survey which lasted for the first three weeks in the month of January this year, the Rs-125 spectrometer was placed about 1 m above the surface cover and the linear energy picked by the detector are within the range of 0.08 MeV and 1.2 MeV, with all the radiation from the terrestrial-based sources being covered. The data acquisition was restricted to the dry season so as to avoid the influence of rain on the local geology of the study area which could influence the mud/clay contents of the soil cover, thereby increasing the levels of the measured radionuclides activity concentrations and gamma radiation dose rates. The data presentation mode were such that the Uranium-238 and Thorium-232 were in part per million, while Potassium-40 was in percentage. The radionuclides data were later converted into $Bqkg^{-1}$ using the conversion factor of IAEA [20].

### 2.2 Descriptive statistics

**Fig. 2:**
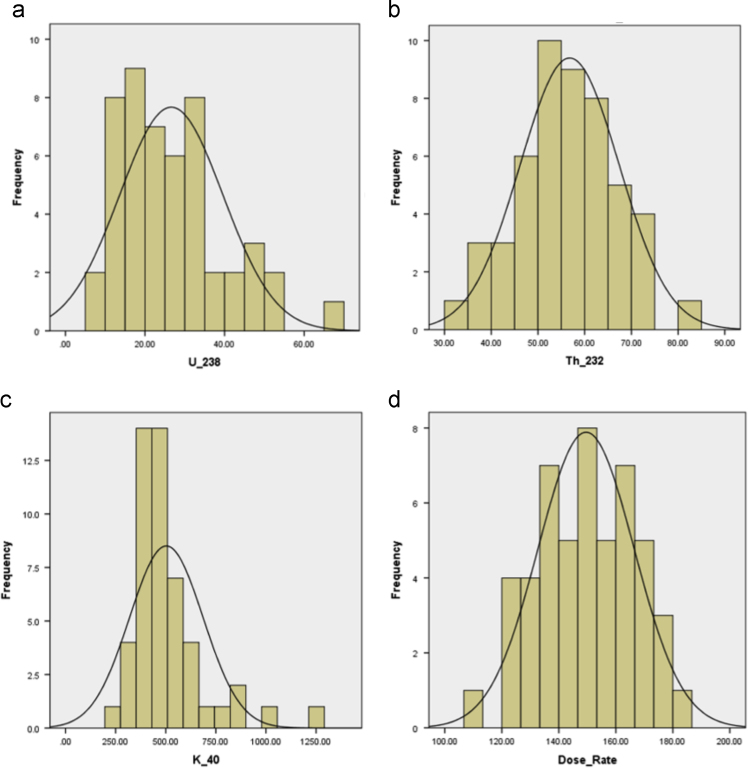
Histogram plots for (a) ^238^U (b) ^232^Th (c) ^40^K (d) gamma-radiation dose rate.

The detailed statistical descriptions which provide basic statistical information about the measured activity concentrations of the radionuclides and the radiation dose rate are presented in Table 2. The histogram plots showing the statistical distribution of each measured radiogenic parameter are shown in Fig. 2.

### 2.3 Correlation analyses

The evaluation of the correlation coefficients using Pearson, Kendall’s tau and Spearman rho techniques was to determine the degree of strength and nature of relationship between the observed radiometric data. The analyses are shown in Table 3 and the numerical values of correlation coefficients among the three different methods appear consistent.
